# Determinants of pain in patients with symptomatic knee osteoarthritis

**Published:** 2016

**Authors:** Behzad Heidari, Karimollah Hajian-Tilaki, Mansour Babaei

**Affiliations:** 1 Mobility Impairment Research Center, Babol University of Medical Sciences, Babol, Iran.; 2 Department of Social Medicine, Babol University of Medical Sciences, Babol, Iran.; 3 Department of Internal Medicine, Ayatollah Rouhani Hospital Division of Rheumatology, Babol University of Medical Sciences, Babol, Iran

**Keywords:** Knee osteoarthritis, Pain, Determinants, Synovitis, C-reactive protein, Structural lesions

## Abstract

**Background:**

Several factors are associated with the development or exacerbation of pain in knee osteoarthritis (KOA). In this study, we reviewed this context based on relevant studies.

**Methods:**

Recent published studies which have addressed the relationship between pain and KOA were summarized.

**Results:**

Correlates of the clinical, demographic features, laboratory tests and abnormalities on radiographic as well as magnetic resonance imaging (MRI) with the knee pain have been discussed. The results indicated that many factors such as synovitis, synovial effusion, obesity, as well as structural lesions determined by MRI or radiographic examination, serum cytokines, inflammatory markers are determinants of pain in KOA.

**Conclusion:**

This context requires further investigations for identification of additional factors which initiate pain in asymptomatic KOA

Knee osteoarthritis (KOA) is characterized by pain and limitation in range of knee joint motion with resultant disability in a substantial proportion of elderly subjects.(1) Pain is the most common clinical presentation of KOA, typically exacerbates by activity and relieves by rest .Persistent pain at rest profoundly affects the quality of life and daily physical activity (1). Pain itself is a risk factor for the development of future functional decline and increasing pain is a predictor of physical functional limitation and disability (2). Nonetheless a proportion of patients with bilateral radiographic KOA present with unilateral knee pain and many asymptomatic subjects have osteoarthritic radiographic changes. On the contrary, a number of patients with knee pain suggestive of clinical KOA, have no radiographic findings (1). 

These observations indicate that some risk factors may have a contributive role in the development as well as exacerbation of pain in KOA (1, 3). The association of KOA with several clinical, demographic, structural and laboratory parameters such as age, sex, obesity, physical activity, synovitis / synovial effusion, subchondral bone lesions, muscle weakness, vitamin D deficiency has been shown (1-3). However, the prevalence and contribution of these factors in the development of pain vary according to patient characteristics and disease status. It was shown that increasing pain along with reduced range of motion, decreased muscle strength, higher morbidity count, higher age, longer disease duration are the predictors of future functional impairment and limitation of activity (2, 4). 

Treatment of KOA is targeted to suppress pain and improve functional outcome. Therefore, information on risk factors of worsening of pain and decline in functional limitation can be used to construct treatment. This study aimed to present a concise information regarding associated factors of pain in patients with KOA (table 1) provided from recent relevant published studies which have addressed the risk factors of pain in KOA.

**Table 1:** Associated factors of pain in patients with knee osteoarthritis

Synovitis and synovial effusion
Vitamin D deficiency
Muscle weakness
Obesity
Female gender
Physical activity
Psychological factors
Joint abnormalities
Magnetic resonance imaging findings
Radiographic changes
Cytokines and inflammatory markers

**Synovitis in KOA: **Recent investigations have found the existence of inflammatory process in several chronic medical conditions as well as osteoarthritis (5-7). Synovial inflammation plays a critical role in the development of symptoms and structural changes in KOA (1, 8-11). In the early stages of KOA synovitis is localized to the regions with chondral defects but at later stages inflammation becomes diffuse and chronic (10, 11). 

Arthroscopic examination reveals chondropathy, synovial infiltration of macrophage and proliferation of blood vessels (11). In osteoarthritic patients without radiographic changes in knee joint, synovitis is observed in 55% of cases (12). In the Multicenter Osteoarthritis Study (MOST) 80% of KOA patients with moderate pain had synovitis (13). In a 1-year longitudinal study of patients with tibiofemoral osteoarthritis with mean age of 61 years, synovial abnormalities were observed in 50% (14). Almost all patients with advanced osteoarthritis who were considered for surgical treatment had synovitis (15). 

**Diagnosis of synovitis: **Definitive diagnosis of synovitis requires biopsy of the synovium during arthroscopic examination (10, 11). However, all patients do not require arthroscopy and thus in suspected cases noninvasive methods such as clinical examination and imaging techniques like ultrasoound particularly magnetic resonance image (MRI) are commonly used to document synovitis. Clinically, the presence of joint pain at rest especially night pain, stiffness, joint effusion indicates synovitis (1, 3). In suspected cases, serum and synovial fluid examination are also helpful to differentiate KOA from other inflammatory arthritis like rheumatoid arthritis (16, 17). In patients with mild or moderate KOA, synovial abnormalities can be detected by using MRI or ultrasound techniques at earlier stages with higher sensitivity than clinical examination even in asymptomatic patients (8, 9, 18-20).

In a study of patients with history of knee pain, a third of cases had synovial thickening or suprapatellar effusion suggestive of synovitis (9). Almost half of patients with KOA show synovial hypertrophy or joint effusion in ultrasounographic examination (21). MRI yields greater sensitivity in detecting joint abnormalities and thus higher proportion of KOA can be recognized by MRI examination. In particular, synovial abnormalities found in MRI show greater correlation with arthroscopic and histologic findings (8, 18, 21). In a study of patients with symtomatic KOA, the types and the frequency of abnormalities seen in MRI examination were synovial thickening, joint effusion, osteophytes and subchondral bone edema which were found in 73%, 60%, 67% and 65% respectively, whereas the respective values in asymptomatic KOA were 0% and 7%, 12% and 7% (8).

**Synovitis and pain: **Like rheumatoid arthritis, osteoarthritis is also associated with inflammation which presents as synovitis. Synovitis in KOA is associated with pain and cartilage loss (13). The association of pain and synovitis in KOA has been shown in several studies (13, 22, 23). The relationship between specific tissue lesions and pain in KOA was show by Torres et al. In this study, synovitis /synovial effusion as well as MRI abnormalities such as bone marrow lesions, subchondral bone attrition and meniscal tears were associated with severity of pain (22). A similar association of pain and synovitis /joint effusion has been reported in KOA with and without radiographic changes (23). In the Multicenter Osteoarthritis Study, pain in patients with synovitis was 9.2-fold greater than those without synovitis even in subjects without radiographic evidence of KOA (13). Nonetheless, in a study by Hill et al., synovial effusion was not associated with pain but changes in synovitis was positively correlated with pain (24). In a study of patients with early KOA,patients without synovitis had no pain (12). Even in patients without KOA, the presence of synovitis causes pain.This was shown in patients with miniscal tears undergoing arthroscopic examination. In this study, patients with preoperative pain had synovitis, and the association was independent to age, sex and severity of cartilage injuries (25).

**Vitamin D and Pain: **Vitamin D deficiency is prevalent in general population as well as patients with musculoskeletal diseases presenting to rheumatology clinics (26-30). Vitamin D deficiency is associated with pain in musculoskeletal diseases including osteoarthritis (27, 30-35).

Low levels of serum vitamin D is a risk factor for the development and worsening of knee pain (34) as well as a predictor for incident KOA (27). Vitamin D deficiency contributes to the development of clinical symptoms and severity of pain through sensitization to pain. Obese vitamin D deficient subjects are at greater risk of pain (31, 36, 37). Compared to vitamin D sufficient subjects, those with moderate to severe vitamin D deficiency are at higher risk of postoperative pain (38). There is a dose-response pattern of relationship between vitamin D deficiency and bone pain/ bone tenderness (35). There is an independent association between vitamin D deficiency and nonspecific bone pain, low back pain, and unexplained arthralgia (26, 30, 32). Normalization of vitamin D deficiency decreases knee pain in patients with KOA (39).

**Muscle strength and knee pain: **Muscle weakness has a significant contribution in the development and progression of KOA (1, 40-42). In KOA, the quadriceps muscle strength is lower than healthy controls (40).

 There is an association between quadriceps muscle weakness and pain as well as disability in KOA independent to radiographic changes (43, 44). In KOA, pain correlates negatively with quadriceps muscle strength and strengthening of quadriceps muscle reduces pain (39, 45).Thus, in these patients muscle strengthening is a target of treatment for relieving pain and improving physical performance (46, 47).

**Obesity, metabolic syndrome and pain: **Obesity and overweight which are prevalent among general popoulation even in young adults and children are strongly associated with onset and exacerbation of pain as well as development of disability in KOA (48-51) whereas weight loss decreases the pain as well as the risk of symptomatic KOA (52). Furtheremore, obesity is a risk factor for the development and progression of KOA (1, 3). In KOA, pain correlates positively with BMI and thus, persons with higher BMI have more severe pain (53). In a cross-sectional study of people with mean age of 58 years, obesity was associated with increased risk of joint pain in hips, knees, ankles and feet (52). In a study by Maddah et al., coexistence of metabolic syndrome with KOA was associated with greater pain and patients with more components of metabolic syndrome had higher Western Ontario and McMaster University Osteoarthritis Index (WOMAC) pain score independent to age and BMI (54). Inasmuchas, abdominal obesity is a major component of metabolic syndrome (55).However, one major component of metsbolic syndrome is abdominal obesity (55).

**Female gender and pain: **Several painful musculoskeletal conditions are more frequent in women than men indicating that women are at greater risk of central sensitization than men (56). Proportion of women with symptomatic KOA is significantly higher than men and radiographic features of KOA are more prevalent in women than in men (57). Prior to total knee replacement surgery, women with KOA have more pain and greater functional impairment than men (58). In a study of elderly community residents with radiographic KOA, women had worse mean WOMAC and SF-36 scores than men with similar radiographic changes (56). In patellofemoral osteoarthritis, women report greater knee pain than men irrespective to severity of radiographic findings (59). In participants of the Rotterdam Study, female gender was a risk factor for pain (60). The association between quadriceps weakness and pain in women is stronger than men. This may partly explain higher susceptibility of women to pain (61). 

**Physical activity and pain: **Influence of physical activity on KOA is not clear and data regarding the harmful effect of different types and intensity of exercise programs on KOA are insufficient (62). Nonetheless, in advanced KOA physical activity aggravates knee pain (63), whereas regular swimming exercise reduces pain and improves muscle strength and functional capacity (64).

The results of a systematic review showed that physical activity increases osteophytes but decreases cartilage lesions (65). Depending on preexisting anatomical changes and type, or duration of activities, physical activity may affect knee or hip joints differently (66). Individuals with history of heavy physical activities show greater joint abnormalities versus those with lower physical activities who have less tibiofemoral or patellofemoral OA (67). High moderate to vigorous activity in subjects with mild knee pain but without radiographic features of KOA will increase bone marrow lesions as well as medial meniscal injuries (68). Also KOA prolong duration of moderate to vigorous daily physical activity increases pain and decreases functional capacity (69). In a longitudinal study, high level of leisure time physical activity increased the risk of total knee replacement surgery in women aged <45 years old but not in men (70). However, the results of a systematic review showed that in older adults with knee pain, long-term physical activity over 3-30 months was safe and did not increase pain or total knee replacement surgery, particularly increasing the level of physical activity reduced disease progression, decreased total knee replacement surgery (71). Similarly, regular to moderate physical activities exerts preventive and therapeutic effect (72). Nevertheless, absence of mechanical stimulation as seen in subjects with spinal injuries results in cartilage thinning (69).

**Psychological and sociodemographic factors and pain: **Psychosocial factors contribute to the development and progression of musculoskeletal pain and disability. In patients with KOA, the prevalence of psychiatric morbidities are higher than expected (73), and these patients have higher number of painful sites in other parts of the body (74). Depression is strongly linked to pain specifically in patients with severe osteoarthritis (75). Presence of depression increases the risk of self-reported knee pain irrespective to severity of radiographic changes. Even in patients with minimal or moderate radiographic changes particularly middle-aged women and elderly subjects, the presence of depression increases pain (76). Furthermore, cognitive factors but not behavioral factors have also a role in the development of pain in osteoarthritis (77). In a longitudinal study with 12-months postoperative follow-up period, postsurgical pain both at rest and activity was higher in depressed patients. In addition, knee function was lower prior to and after surgery and the prevalence of dissatisfaction was higher (78). Socioeconomic factors affect osteoarthritis symptoms and treatment results. Subjects of higher socioeconomic levels have less pain and higher functional capacity prior to total knee replacement surgery (79).

**MRI abnormalities and pain: **MRI is a sensitive method for visualization of various joint structures including cartilage, bone marrow, synovium, meniscal and ligamental pathology in both tibiofemoral and patellofemoral joints (3, 8, 23, 80), whereas, conventional anteroposterior radiographs have no ability to disclose patellofemoral compartment, ligaments or synovium (80). In a study of patients with definite knee ostephytes in radiograph, all MRI abnormalities such as subarticular bone attrition, bone marrow lesions, synovitis/effusion, and meniscal tears were significantly associated with knee pain (22). However, in another case-control study, only the presence of large bone edema differentiated painful KOA from asymptomatic KOA or healthy controls (81). The results of a systematic review of 22 studies showed a significant association between bone marrow lesions as well as synovitis/effusion with pain. Bone marrow lesion increased the odds of pain 2 to 5-folds and synovitis increased the pain by odds of 3.2 to 10 (82). In a cross-sectional study of 904 randomly selected subjects with the average age of 62.4 years, patellar bone marrow lesions were consistently associated with knee pain particularly going up/down stairs (83). Association between MRI findings and knee pain was also observed in Michigan Study of Women's Health across the Nation (84).

**Radiologic abnormalities and pain: **Knee pain is a vague symptom of KOA and its association with radiographic abnormalities varies according to extent of radiographic changes and radiographic view. The results of studies which have addressed the relation between pain and radiographic changes in KOA are controversial. This may be attributed to types, severity and the prevalence radiographic abnormalities (60, 85). The clinical and radiographic features of KOA are not usually concordant with KOA symptoms. 

The results of a systematic review reveals that only 15-76 % of subjects with knee pain had radiographic features of KOA and only 15-81% of subjects with radiographic OA had knee pain (85). 

 In a study of 2282 elderly Japanese subjects aged > 60 years, the association between radiographic changes and pain varied from none to strong. Nonetheless, the presence of severe radiographic changes were strongly associated with pain (85). 

In Baltimore Longitudinal Study of Aging, over a 5-year follow-up period, all measures of radiographic severity were directly correlated with knee pain (86). In a study of the elderly Japanese population-based cohorts, joint space narrowing was strongly associated with pain especially in men (87). In another population-based study of 250 subjects, osteophytes was strongly associated with knee pain, whereas the presence or absence of joint space narrowing was not associated with pain (88). Radiographic abnormalities can reduce muscle strength and result in pain. **(**3). An inverse association between quadriceps muscle strength and radiographic progression especially in patients with radiographic grades of 0 and 1 has been reported (89).

**Inflammatory markers and pain: **Chronic systemic inflammation and local synovitis are source of pain in KOA (25). Production of local inflammatory cytokines and nerve growth factor in the synovium promote pain. In the early stage of osteoarthritis, synovium produces cytokines such as interleukin-1, TNF-alpha and IL-6. These cytokines cause increased production of serum CRP and serum amyloid A (8). In addition, serum markers and surrogates of synovial fluid can be used as markers for diagnosis, prognosis, or evaluation of disease, severity or response to treatment (16, 17, 87, 90). 

In advanced osteoarthritis, synovitis correlates with both plasma CRP and synovial fluid IL-6 (91). In KOA serum CRP, IL-6, and IL-10 are higher than healthy controls. These markers correlate positively with pain as well as radiographic abnormalities (92-94). Furthermore, serum CRP correlates with synovial fluid CRP as well as severity of osteoarthritis (92). Patients with higher levels of serum CRP have greater postoperative functional impairment (95). Moreover pain in KOA deteriorates by increasing serum levels of both serum CRP, TNF-a and IL-6 (96).

 In conclusion, a high proportion of patients with KOA are asymptomatic inspite of radiographic evidence of osteoarthritis. Nonetheless pain occurs in a significant number of these patients which may increase with age. Several factors like synovitis, joint effusion, and structural changes as seen in radiographs or MRI are associated with pain. As a consequence many associated factors of pain such as obesity, muscle weakness, vitamin D deficiency are modifiable. Therefore, in approaching patients with KOA, identification of the associated factors is important for the establishment of treatment and prevention measures.

## Citation:

Heidari B, Hajian-Tilaki K, Babaei M. Determinants of pain in patients with symptomatic knee osteoarthritis. Caspian J Intern Med 2016; 7(3): 153-161.

## References

[1] Heidari B (2011). Knee osteoarthritis prevalence, risk factors, pathogenesis and features: Part I. Caspian J Intern Med.

[2] Pisters MF, Veenhof C, van Dijk GM (2012). The course of limitations in activities over 5 years in patients with knee and hip osteoarthritis with moderate functional limitations: risk factors for future functional decline. Osteoarthritis Cartilage.

[3] Heidari B (2011). Knee osteoarthritis diagnosis, treatment and associated factors of progression: Part II. Caspian J Intern Med.

[4] Dekker J1, van Dijk GM, Veenhof C (2009). Risk factors for functional decline in osteoarthritis of the hip or knee. Curr Opin Rheumatol.

[5] Heidari B (2012). The importance of C-reactive protein and other inflammatory markers in patients with chronic obstructive pulmonary disease. Caspian J Intern Med.

[6] Firouzjahi A, Monadi M, Karimpoor F (2013). Serum C-reactive protein level and distribution in chronic obstructive pulmonary disease versus healthy controls: a case-control study from Iran. Inflammation.

[7] Heidari B (2013). C-reactive protein and other markers of inflammation in hemodialysis patients. Caspian J Intern Med.

[8] Loeuille D, Chary-Valckenaere I, Champigneulle J (2005). Macroscopic and microscopic features of synovial membrane inflammation in the osteoarthritic knee: correlating magnetic resonance imaging findings with disease severity. Arthritis Rheum.

[9] Kumm J, Tamm A, Lintrop M, Tamm A (2009). Association between ultrasonographic findings and bone/cartilage biomarkers in patients with early-stage knee osteoarthritis. Calcif Tissue Int.

[10] Ene R, Sinescu RD, Ene P, Cirstoiu MM, Cirstoiu FC (2015). Synovial inflammation in patients with different stages of knee osteoarthritis. Rom J Morphol Embryol.

[11] Benito MJ, Veale DJ, FitzGerald O, van den Berg WB, Bresnihan B (2005). Synovial tissue inflammation in early and late osteoarthritis. Ann Rheum Dis.

[12] Myers SL, Brandt KD, Ehlich JW (1990). Synovial inflammation in patients with early osteoarthritis of the knee. J Rheumatol.

[13] Baker K, Grainger A, Niu J (2010). Relation of synovitis to knee pain using contrast-enhanced MRIs. Ann Rheum Dis.

[14] Ayral X, Pickering EH, Woodworth TG (2005). Synovitis: a potential predictive factor of structural progression of medial tibiofemoral knee osteoarthritis-- results of a 1 year longitudinal arthroscopic study in 422 patients. Osteoarthritis Cartilage.

[15] Haywood L, McWilliams DF, Pearson CI (2003). Inflammation and angiogenesis in osteoarthritis. Arthritis Rheum.

[16] Heidari B, Firouzjahi A, Heidari P, Hajian K (2009). The prevalence and diagnostic performance of anti-cyclic citrullinated peptide antibody in rheumatoid arthritis: the predictive and discriminative ability of serum antibody level in recognizing rheumatoid arthritis. Ann Saudi Med.

[17] Heidari B, Abedi H, Firouzjahi A, Heidari P (2010). Diagnostic value of synovial fluid anti-cyclic citrullinated peptide antibody for rheumatoid arthritis. Rheumatol Int.

[18] Fernandez-Madrid F, Karvonen RL, Teitge RA (1995). Synovial thickening detected by MR imaging in osteoarthritis of the knee confirmed by biopsy as synovitis. Magn Resn Imaging.

[19] Loeuille D, Chary-Valckenaere I, Champigneulle J (2005). Macroscopic and microscopic features of synovial membrane inflammation in the osteoarthritic knee: correlating magnetic resonance imaging findings with disease severity. Arthritis Rheum.

[20] Loeuille D, Rat AC, Goebel JC (2009). Magnetic resonance imaging in osteoarthritis: which method best reflects synovial membrane inflammation? Correlations with clinical, macroscopic and microscopic features. Osteoarthritis Cartilage.

[21] Zeng C, Li YS, Lei GH (2015). Synovitis in knee osteoarthritis: a precursor or a concomittant feature?. Ann Rheum Dis.

[22] Torres L, Dunlop DD, Peterfy C (2006). The relationship between specific tissue lesions and pain severity in persons with knee osteoarthritis. Osteoarthritis Cartilage.

[23] Ai F1, Yu C, Zhang W (2010). MR imaging of knee osteoarthritis and correlation of findings with reported patient pain. Huazhong Univ Sci Technolog Med Sci.

[24] Hill CL, Hunter D, Niu J (2007). Synovitis detected on magnetic resonance imaging and its relation to pain and cartilage loss in knee osteoarthritis. Ann Rheum Dis.

[25] Scanzello CR, McKeon B, Swaim BH (2011). Synovial inflammation in patients undergoing arthroscopic meniscectomy: molecular characterization and relationship to symptoms. Arthritis Rheum.

[26] Heidari B, Javadian Y, Heidari P (2014). Vitamin D deficiency is associated with nonspecific low back pain in young women, a case-control study. Br J Med Med Res.

[27] Heidari B, Heidari P, Samari E, Ramzanian Jalali M (2014). Prevalence of vitamin D deficiency in common musculoskeletal conditions. J Babol Univ Sci.

[28] Heidari B, Heidari P, Hajian Tilaki K (2013). High prevalence of vitamin d deficiency in women presenting to rheumatology clinic in north of Iran: an inverse relation with age. J Womens Health Care.

[29] Heidari B, Haj Mirghassemi M (2012). Seasonal variations in serum vitamin D according to age and sex. Caspian J Intern Med.

[30] Heidari B, Heidari P, Hajian Tilaki K (2011). Association between serum vitamin D deficiency and knee osteoarthritis. Int Orthop.

[31] Laslett LL, Quinn S, Burgess JR (Ann Rheum Dis. 2014). et al. Moderate vitamin d deficiency is associated with changes in knee and hip pain in older adults: a 5-year longitudinal study.

[32] Heidari B, Heidari P, Tilaki KH (2014). Relationship between unexplained arthralgia and vitamin D deficiency: a case control study. Acta Med Iran.

[33] Heidari B, Hajian-Tilaki K, Heidari P (2012). The status of serum vitamin D in patients with rheumatoid arthritis and undifferentiated inflammatory arthritis compared with controls. Rheumatol Int.

[34] Al-Jarallah KF, Shehab D, Al-Awadhi A (2011). Are 25(OH)D levels related to the severity of knee osteoarthritis and function?. Med Prin Pract.

[35] Heidari B, Shirvani JS, Firouzjahi A, Heidari P, Hajian-Tilaki KO (2010). Association between nonspecific skeletal pain and vitamin D deficiency. Int J Rheum Dis.

[36] Glover TL, Horgas AL, Fillingim RB, Goodin BR (2015). Vitamin D status and pain sensitization in knee osteoarthritis: a critical review of the literature. Pain Manag.

[37] Glover TL, Goodin BR, Horgas AL (2012). Vitamin D, race, and experimental pain sensitivity in older adults with knee osteoarthritis. Arthritis Rheum.

[38] Lee A, Chan SK, Samy W, Chiu CH, Gin T (2015). Effect of hypovitaminosis d on postoperative pain outcomes and short-term health-related quality of life after knee arthroplasty: a cohort study. Medicine (Baltimore).

[39] Heidari B, Javadian Y, Babaei M, Yousef-Ghahari B (2015). Restorative effect of vitamin d deficiency on knee pain and quadriceps in knee osteoarthritis. Acta Med Iran.

[40] Heidari B, Javadian Y, Akbari R (2016). Quadriceps muscle strength in patients with knee osteoarthritis. J Zanjan Univ Med Sci Health Serv.

[41] Alnahdi AH, Zeni JA, Snyder-Mackler L (2012). Muscle impairments in patients with knee osteoarthritis. Sports Health.

[42] Heidari B (2012). Muscle strength, vitamin D deficiency and knee osteoarthritis. J Babol Univ Med Sci.

[43] Muraki S, Akune T, Teraguchi M (2015). Quadriceps muscle strength, radiographic knee osteoarthritis and knee pain: the ROAD study. BMC Musculoskelet Disord.

[44] Amin S, Baker K, Niu J (2009). Quadriceps strength and the risk of cartilage loss and symptom progression in knee osteoarthritis. Arthritis Rheum.

[45] Javadian Y, Adabi M, Heidari B, Babaei M, Firouzjahi A, Ghahhari BY, Hajian-Tilaki K (2016). Quadriceps correlates with serum vitamin D and knee pain in knee osteoarthritis. Clin J Pain.

[46] Lack S, Barton C, Sohan O, Crossley K, Morrissey D (2015). Proximal muscle rehabilitation is effective for patellofemoral pain: a systematic review with meta-analysis. Br J Sports Med.

[47] Juhl C, Christensen R, Roos EM, Zhang W, Lund H (2014). Impact of exercise type and dose on pain and disability in knee osteoarthritis: a systematic review and meta-regression analysis of randomized controlled trials. Arthritis Rheumatol.

[48] Hajian‐Tilaki K, Heidari B (2007). Obesity Prevalence of obesity, central obesity and the associated factors in urban population aged 20–70 years, in the north of Iran: a population‐based study and regression approach. Obes Rev.

[49] Hajian-Tilaki K, Heidari B (2012). Prevalences of overweight and obesity and their association with physical activity pattern among Iranian adolescents aged 12-17 years. Public Health Nutr.

[50] Hajian-Tilak K, Heidari B (2013). Childhood obesity, overweight, socio demographic and life style determinants among preschool children in Babol, Northern Iran. Iran J Public Health.

[51] Webb R, Brammah T, Lunt M (2004). Opportunities for prevention of ‘clinically significant knee pain: results from a population‐based cross sectional survey. J Public Health.

[52] Adamson J, Ebrahim S, Dieppe P, Hunt K (2006). Prevalence and risk factors for joint pain among men and women in the West of Scotland Twenty-07 study. Ann Rheum Dis.

[53] Rogers MW, Wilder FV (2008). The association of BMI and knee pain among persons with radiographic knee osteoarthritis: a cross-sectional study. BMC Musculoskeletal Disord.

[54] Maddah S, Mahdizadeh J (2015). Association of metabolic syndrome and its components with knee osteoarthritis. Acta Med Iran.

[55] Hajian-Tilaki K, Heidari B, Firouzjahi A (2014). Prevalence of metabolic syndrome and the association with socio-demographic characteristics and physical activity in urban population of Iranian adults: a population-based study. Diabetes Metab Syndr.

[56] Cho HJ, Chang CB, Yoo JH, Kim SJ, Kim TK (2010). Gender differences in the correlation between symptom and radiographic severity in patients with knee osteoarthritis. Clin Orthop Relat Res.

[57] Felson DT, Naimark A, Anderson J (1987). The prevalence of knee osteoarthritis in the elderly. The Framingham osteoarthritis study. Arthritis Rheum.

[58] Tonelli SM, Rakel BA, Cooper NA, Angstom WL, Sluka KA (2011). Women with knee osteoarthritis have more pain and poorer function than men, ut similar physical activity prior to total knee replacement. Biol Sex Diff.

[59] Glass N, Segal NA, Sluka KA (2014). Examining sex differences in knee pain: the multicenter osteoarthritis study. Osteoarthritis Cartilage.

[60] Schiphof D, Kerkhof HJ, Damen J (2013). Factors for pain in patients with different grades of knee osteoarthritis. Arthritis Care Res (Hoboken).

[61] Glass NA, Torner JC, Frey Law LA (2013). The relationship between quadriceps muscle weakness and worsening of knee pain in the MOST cohort: a 5-year longitudinal study. Osteoarthritis Cartilage.

[62] Regnaux JP, Lefevre-Colau MM, Trinquart L (2015). High-intensity versus low-intensity physical activity or exercise in people with hip or knee osteoarthritis. Cochrane Database Syst Rev.

[63] Liu SH, Driban JB, Eaton CB (2016). Objectively measured physical activity and symptoms change in knee osteoarthritis. Am J Med.

[64] Alkatan M, Baker JR, Machin DR (2016). Improved function and reduced pain after swimming and cycling training in patients with osteoarthritis. J Rheumatol.

[65] Urquhart DM, Tobing JF, Hanna FS (2011). What is the effect of physical activity on the knee joint? A systematic review. Med Sci Sports Exerc.

[66] Wang Y, Simpson JA, Wluka AE (2011). Is physical activity a risk factor for primary knee or hip replacement due to osteoarthritis? A prospective cohort study. J Rheumatol.

[67] Kujala UM, Kettunen J, Paananen H (1995). Knee osteoarthritis in former runners, soccer players, weight lifters, and shooters. Arthritis Rheum.

[68] Murphy SL, Schepens Nimiec SL, Lyden AK, Kratz AL (2016). Pain, fatigue, and physical activity in osteoarthritis. The moderating effect of pain-and fatigue -related activity interference. Arch Phus Med Rehabil.

[69] Kretzschmar M, Lin W, Nardo L (2015). Association of physical activity measured by accelerometer, knee joint abnormalities, and cartilage T2 measurements obtained from 3t magnetic resonance imaging: data from the osteoarthritis initiative. Arthritis Care Res (Hoboken).

[70] Lin W, Alizai H, Joseph GB (2013). Physical activity in relation to knee cartilage T2 progression measured with 3 T MRI over a period of 4 years: data from the Osteoarthritis Initiative. Osteoarthritis Cartilage.

[71] Fransen M, McConnell S, Harmer AR (2015). Exercise for osteoarthritis of the knee: a Cochrane systematic review. Br J Sports Med.

[72] Johnsen MB, Hellevik AI, Baste V (2016). Leisure time physical activity and the risk of hip or knee replacement due to primary osteoarthritis: a population based cohort study (The HUNT Study). BMC Musculoskelet Disord.

[73] Wong LY, Yiu RL, Chiu CK (2015). Prevalence of psychiatric morbidity in Chinese subjects with knee osteoarthritis in a Hong Kong orthopaedic clinic. East Asian Arch Psychiatry.

[74] Dave AJ, Selzer F, Losina E (2015). Is there an association between whole-body pain with osteoarthritis-related knee pain, pain catastrophizing, and mental health?. Clin Orthop Relat Res.

[75] Hernández C, Díaz-Heredia J, Berraquero ML (2015). Pre-operative predictive factors of post-operative pain in patients with hip or knee arthroplasty: a systematic review. Reumatol Clin.

[76] Han HS, Lee JY, Kang SB, Chang CB (2015). The relationship between the presence of depressive symptoms and the severity of self-reported knee pain in the middle aged and elderly. Knee Surg Sports Traumatol Arthrosc.

[77] Urquhart DM, Phyomaung PP, Dubowitz J (2015). Are cognitive and behavioral factors associated with knee pain? A systematic review. Semin Arthritis Rheum.

[78] Bierke S, Häner M, Petersen W (2016). Influence of somatization and depressive symptoms on the course of pain within the first year after uncomplicated total knee replacement: a prospective study. Int Orthop.

[79] Feldman CH, Dong Y, Katz JN, Donnell-Fink LA, Losina E (2015). Association between socioeconomic status and pain, function and pain catastrophizing at presentation for total knee arthroplasty. BMC Musculoskelet Disord.

[80] Conaghan PG1, Felson D, Gold G (2006). MRI and non-cartilaginous structures in knee osteoarthritis. Osteoarthritis Cartilage.

[81] Hayes CW1, Jamadar DA, Welch GW (2005). Osteoarthritis of the knee: comparison of MR imaging findings with radiographic severity measurements and pain in middle-aged women. Radiology.

[82] Yusuf E, Kortekaas MC, Watt I, Huizinga TW, Kloppenburg M (2011). Do knee abnormalities visualised on MRI explain knee pain in knee osteoarthritis? A systematic review. Ann Rheum Dis.

[83] Wang J, Antony B, Zhu Z (2015). Association of patellar bone marrow lesions with knee pain, patellar cartilage defect and patellar cartilage volume loss in older adults: a cohort study. Osteoarthritis Cartilage.

[84] Sowers M, Karvonen-Gutierrez CA, Jacobson JA, Jiang Y, Yosef M (2011). Associations of anatomical measures from MRI with radiographically defined knee osteoarthritis score, pain, and physical functioning. J Bone Joint Surg Am.

[85] Bedson J, Croft PR (2008). The discordance between clinical and radiographicknee osteoarthritis: a systematic search and summary of the literature. BMC Musculoskelet Disord.

[86] Lethbridge CM, Scott-Jr WW, Reichle R (1995). Association of radiographic features of osteoarthritis of the knee with knee pain: Data from the Baltimore longitudinal study of aging. Arthritis Care Res.

[87] Muraki S, Oka H, Akune T (2009). Prevalence of radiographicknee osteoarthritis and its association with knee pain in the elderly of Japanese population-based cohorts: the ROAD study. Osteoarthritis Cartilage.

[88] Cicuttini FM1, Baker J, Hart DJ, Spector TD (1996). Association of pain with radiological changes in different compartments and views of the knee joint. Osteoarthritis Cartilage.

[89] Omori G, Koga Y, Tanaka M (2013). Quadriceps muscle strength and its relationship to radiographic knee osteoarthritis in Japanese elderly. J Orthop Sci.

[90] Heidari B, Heidari P, Tayebi ME (2007). The value of changes in CRP and ESR for predicting treatment response in rheumatoid arthritis. APLAR J Rheumatol.

[91] Pearle AD, Scanzello CR, George S (2007). Elevated high-sensitivity C-reactive protein levels are associated with local inflammatory findings in patients with osteoarthritis. Osteoarthritis Cartilage.

[92] Wolfe F (1997). The C-reactive protein but not erythrocyte sedimentation rate is associated with clinical severity in patients with osteoarthritis of the knee or hip. J Rheumatol.

[93] Jin X, Beguerie JR, Zhang W (2015). Circulating C reactive protein in osteoarthritis: a systematic review and meta-analysis. Ann Rheum Dis.

[94] Zhu Z, Jin X, Wang B (2016). Cross-sectional and longitudinal associations between serum levels of hs-CRP, knee bone marrow lesions and knee pain in patients with knee osteoarthritis. Arthritis Care Res (Hoboken).

[95] Smith JW1, Martins TB, Gopez E (2012). Significance of C-reactive protein in osteoarthritis and total knee arthroplasty outcomes. Ther Adv Musculoskelet Dis.

[96] Stannus OP, Jones G, Blizzard L, Cicuttini FM, Ding C (2013). Associations between serum levels of inflammatory markers and change in knee pain over 5 years in older adults: a prospective cohort study. Ann Rheum Dis.

